# Optimized APPS-tDCS electrode position, size, and distance doubles the on-target stimulation magnitude in 3000 electric field models

**DOI:** 10.1038/s41598-022-24618-3

**Published:** 2022-11-22

**Authors:** Kevin A. Caulfield, Mark S. George

**Affiliations:** 1 Brain Stimulation Laboratory, Department of Psychiatry, Medical University of South Carolina, 67 President Street, 504N, Charleston, SC 29425 USA; 2 Ralph H. Johnson VA Medical Center, Charleston, SC USA

**Keywords:** Biophysical models, Brain-machine interface

## Abstract

Transcranial direct current stimulation (tDCS) is a widely used noninvasive brain stimulation technique with mixed results to date. A potential solution is to apply more efficient stimulation to ensure that each participant receives sufficient cortical activation. In this four-part study, we used electric field (E-field) modeling to systematically investigate the cortical effects of conventional and novel tDCS electrode montages, with the goal of creating a new easily adoptable form of tDCS that induces higher and more focal E-fields. We computed 3000 anatomically accurate, MRI-based E-field models using 2 mA tDCS to target the left primary motor cortex in 200 Human Connectome Project (HCP) participants and tested the effects of: 1. Novel Electrode Position, 2. Electrode Size, and 3. Inter-Electrode Distance on E-field magnitude and focality. In particular, we examined the effects of placing electrodes surrounding the corticomotor target in the anterior and posterior direction (anterior posterior pad surround tDCS; APPS-tDCS). We found that electrode position, electrode size, and inter-electrode distance all significantly impact the cortical E-field magnitude and focality of stimulation (all *p* < 0.0001). At the same 2 mA scalp stimulation intensity, APPS-tDCS with smaller than conventional 1 × 1 cm electrodes surrounding the neural target deliver more than double the on-target cortical E-field (APPS-tDCS: average of 0.55 V/m from 2 mA; M1-SO and bilateral M1: both 0.27 V/m from 2 mA) while stimulating only a fraction of the off-target brain regions; 2 mA optimized APPS-tDCS produces 4.08 mA-like cortical E-fields. In sum, this new optimized APPS-tDCS method produces much stronger cortical stimulation intensities at the same 2 mA scalp intensity. APPS-tDCS also more focally stimulates the cortex at the intended target, using simple EEG coordinate locations and without MRI scans. This APPS-tDCS method is adoptable to any existing, commercially available tDCS device and can be used to ensure sufficient cortical activation in each person. Future directions include testing whether APPS-tDCS produces larger and more consistent therapeutic tDCS effects.

## Introduction

Transcranial direct current stimulation (tDCS) is a widely used form of noninvasive brain stimulation^1^ applied in healthy adults and over 20 neurological or psychiatric diagnoses, including depression^2–4^, post-stroke motor rehabilitation^5,6^, aphasia^7–9^, and anxiety^10,11^. By passing small uniform electrical currents of 1 or 2 mA through anodal and cathodal scalp electrodes, tDCS can open sodium-dependent ion channels in underlying neurons^12^, increasing intracellular calcium concentration^13,14^ and driving long term potentiation (LTP) ^15^. However, despite widespread interest in tDCS and appeal due to its noninvasive^16^, inexpensive^17^, and easily disseminatable nature with potential at-home use^18–22^, there are inconsistent effects of stimulation^23–28^ and no FDA-approved indication for tDCS to date.

A possible reason underlying inconsistent tDCS results is that applying the same 2 mA scalp dose for each person might result in suboptimal amounts of stimulation reaching the cortex in some people due to anatomical variation^29–32^. Converging lines of evidence from cadaver research^29^ and electric field (E-field) modeling suggest that higher cortical E-fields could produce stronger behavioral results, particularly in depression^33^ and working memory^34^. These data support investigating whether higher or individualized amounts of stimulation might improve clinical tDCS effects. Researchers have begun to test the safety and efficacy of higher tDCS intensities up to 4 mA^30,35,36^, with early reports of stronger corticomotor effects compared to 2 mA and sham^35^. These efforts are complemented by researchers developing methods of individualizing tDCS dosing based on E-field modeling^32,37^, and automatic methods of calculating the optimal scalp electrode/coil locations and intensities to produce the chosen cortical E-field magnitude^38–41^ (Table 1).

**Table 1:** Pros and Cons of Different tDCS Stimulation Strategies.

	Conventional 2 mA tDCS	2 mA HD-tDCS	Conventional 4 mA tDCS	Individualized E-Field tDCS	Ideal Method
Set-up	Pad Electrodes	4 × 1 Circular Electrodes	Pad Electrodes	Pad or 4 × 1 Circular Electrodes	Pad Electrodes
Pros	Easy to use with minimal side effects and at-home potential	Focal stimulation at target with minimal off-target effects	Could have stronger or more consistent effects vs. 2 mA	Personalized scalp intensity for same on-target E-field across all patients	Easy to use with higher cortical intensity and lower risk of increased side effects; no MRI scans
Cons	Mixed effects to date	Focality-intensity tradeoff = lower intensity at target; more complex electrode set-up	Greater risk of side effects or improper use at-home; largely untested	Requires MRI scans and computational expertise; lower potential for at-home use	Not applicable

Taken together, there is building support for the use of 4 mA tDCS or individualized E-field dosing to produce stronger tDCS effects, particularly in individuals who may have been underdosed at typical 2 mA stimulation intensities (Table 1). However, limitations of these approaches are that 4 mA tDCS could cause higher incidence of scalp burning or sensations, may be less safe to self-administer, and in the case of E-field dosing, requires MRI scans and electrode placements in non-EEG based locations making it harder to replicate at home (Table 1). In sum, there is an incentive to develop a tDCS approach that produces higher on-target E-fields but does not require MRI scans. This ideal approach would retain the low cost, easily disseminatable, and at-home appeal of tDCS while possibly producing more consistent and stronger behavioral effects (Table 1).

In this four-part study, we performed large-scale E-field modeling in 200 Human Connectome Project (HCP) participants^42^ to computationally test the effects of EEG-based tDCS electrode positioning, size, and inter-electrode distance on E-field magnitude and focality. Our goal was to inform a new, more efficient tDCS approach that might be capable of producing stronger E-fields at the same scalp stimulation intensity. This approach mirrors Datta et al.’s (2009) development of 4 × 1 high definition (HD)-tDCS, in which E-field modeling demonstrated how HD-tDCS produces more focal stimulation prior to human application^43^. Similarly, here we used E-field modeling to provide a theoretical basis for tDCS electrode optimization, building on prior work demonstrating that tDCS electrode positioning matters^44,45^, and that the highest E-field is often midway between electrodes^34,46^. Drawing on this concept, Mikkonen et al. (2020) designed conventional tDCS with electrodes placed with the target in the middle^47^. Other researchers reported edge effects such that the E-field is strongest in the brain regions between the electrodes and lower directly underneath them^48,49^. Taken together, an optimal, efficient electrode montage would produce higher E-fields with higher focality than conventional tDCS (i.e., high on-target and low off-target effects; Table 1). Notably, HD-tDCS and other prior approaches typically have an inverse relationship between E-field magnitude and focality^50,51^, making a combination of both high on-target and low off-target effects appealing and novel. Our goal also extended to basing the electrode placements on EEG coordinates and being compatible with any existing tDCS device, enabling fast and widespread dissemination of this new approach.

In Round 1 modeling, we compared the E-fields produced from conventional electrode montages, and introduced two new electrode placements: Anterior Posterior Pad Surround (APPS)-tDCS, with pad electrodes surrounding the cortical target in the anterior and posterior directions, and Left Right Pad Surround (LRPS)-tDCS, with pad electrodes surrounding the target in the left and right directions. In Round 2 modeling, we systematically tested the effects of electrode size on E-field intensity with the APPS-tDCS montage, while keeping the inter-electrode distance constant. In Round 3, we kept the electrode size constant and systematically altered the inter-electrode distance to determine how distance scales with E-field magnitude and focality. In Round 4, we combined the ‘winning’ electrode positioning, size, and inter-electrode distance from Rounds 1–3 to create an optimized APPS-tDCS set-up. We hypothesized that surrounding the cortical target with smaller electrodes placed at an optimized distance apart would cause the highest E-field magnitudes with relatively high focality (i.e., highest on-target and lowest off-target effects).

## Methods

### Overview

We computed 3000 electric field models in 200 HCP participants, using the same 2 mA stimulation intensity in each model and a within-subjects design (15 paired models per participant). Each model placed electrodes that were intended to target the left motor cortex as a representative brain region that is commonly targeted in tDCS, with the idea that these principles could be translated to other cortical targets in the future. All models used 10–10 EEG-coordinates or a variation of 10–10 coordinates to place electrodes, such that the current findings can be easily implemented in tDCS applications moving forward.

### Participants and Scanning Protocol

We accessed the open access WU-Minn HCP Data Archive^42^, screening for healthy adult participants with T1w and T2w structural MRI scans which we used for E-field modeling. Informed consent was obtained from each participant in IRB-approved protocols across all sites, and all experiments were performed in accordance with ethical guidelines and regulations. Of the 1113 individuals with deidentified structural scans, we randomly selected 200 participants (100 male/100 female). As part of the deidentification process for the public database, only general age ranges are reported in the HCP data. As such, we selected 33F/33 M in the 22–25 range, 34F/34 M in the 26–30 range, and 33F/33 M in the 31–35 range. Structural MRI scans were acquired with Siemens MAGNETOM 3 T scanners and 32-channel head coils. T1w scan parameters were as follows: TR = 2400 ms, TE = 2.14 ms, flip angle = 8°, field of view = 224 mm x 224 mm x 180 mm, voxel size = 0.7mm^3^. T2w scans were acquired at the following parameters: TR = 3200 ms, TE = 565 ms, field of view = 224 mm x 224 mm x 180 mm, voxel size = 0.7mm^3^. Full imaging parameters are available at the HCP database, *Appendix I: HCP scan protocols*.

### Electric field modeling

**Figure 1:**
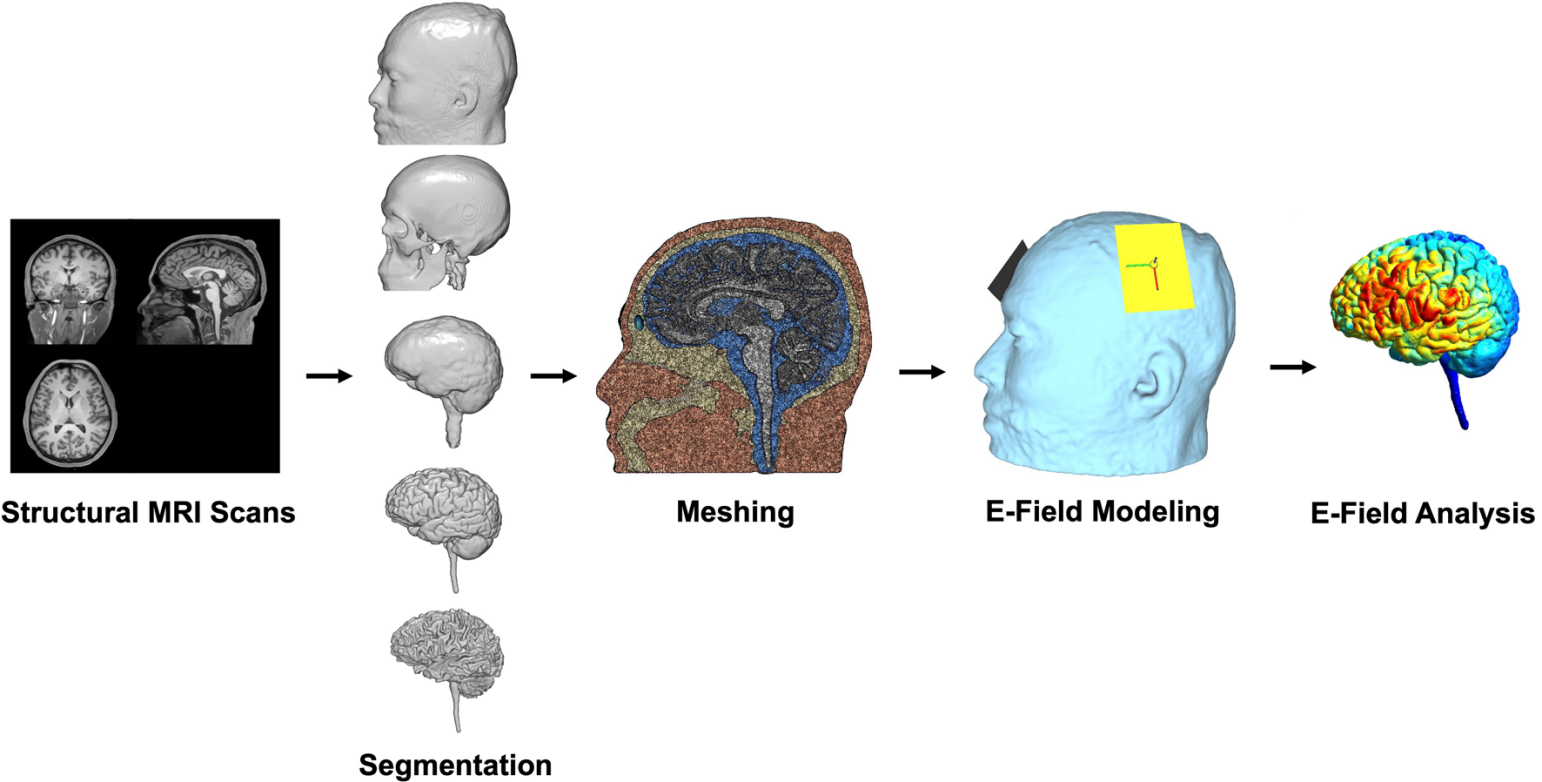
E-Field Modeling Pipeline. E-field modeling entails five main steps. First, the researcher acquires structural MRI scans (i.e., T1w and T2w scans). Next, the structural MRI scans are segmented into different components (top to bottom: skin, bone, cerebrospinal fluid, grey matter, and white matter). Third, the tissue segmentations are combined into a 3D volumetric mesh to create an anatomically accurate head model. Fourth, E-field modeling uses experimentally determined tissue conductivity values to simulate how much electric current applied at the scalp reaches the cortex. Displayed is an example in which 7 × 5 cm electrodes were placed over the left primary motor cortex (M1) and right supraorbital (SO) cortex. Lastly, the researcher performs E-field analyses. See Fig. 2 for detailed description of E-field analyses in this study, which included a region of interest (ROI) analysis, 99th percentile threshold analysis, and focality analysis, defined as a measure of volume stimulated at or above the 50th percentile E-field.

For segmentation and meshing of T1w and T2w structural MRI scans^52,53^, we used headreco^54^. Headreco utilizes SPM12 and CAT12 to segment each scan into skin, skull, cerebrospinal fluid, white matter, grey matter, and eyes (Fig. 1). Following segmentation, headreco meshed the tissue layers together using the finite element method (FEM). We visually evaluated the integrity of the FEM mesh to ensure that the tissue segmentations were clearly delineated between layers. We assigned default tissue conductivity values to each tissue layer: skin: 0.465S/m, bone: 0.01S/m, cerebrospinal fluid: 1.654S/m, grey matter: 0.275S/m, white matter: 0.126S/m, and eyeballs: 0.50S/m, and with a mesh density of 0.5 nodes per mm^2^. For E-field modeling, we used SimNIBS 3.2.3^55^, which calculates electric fields using Dirichlet boundary conditions on the electrode surfaces and homogenous Neumann boundary conditions elsewhere in the mesh^56^.

### Electrode montages

We performed four rounds of E-field modeling with the goal of optimizing electrode position, size, and distance based on 10–10 EEG coordinates. Each model simulated 2 mA tDCS targeting the left primary motor cortex (M1). 10–10 EEG locations were automatically determined by headreco, which transforms EEG 10–10 electrode locations defined in MNI space to subject space using a non-linear transform, then projecting the XYZ positions to the scalp^55^. Compared to manual fiducial determination of the 10–10 EEG coordinates, the automated headreco pipeline produced minimal errors, with an average deviation of 4.9 mm and with all locations underneath 10 mm^55^.

**Figure 2:**
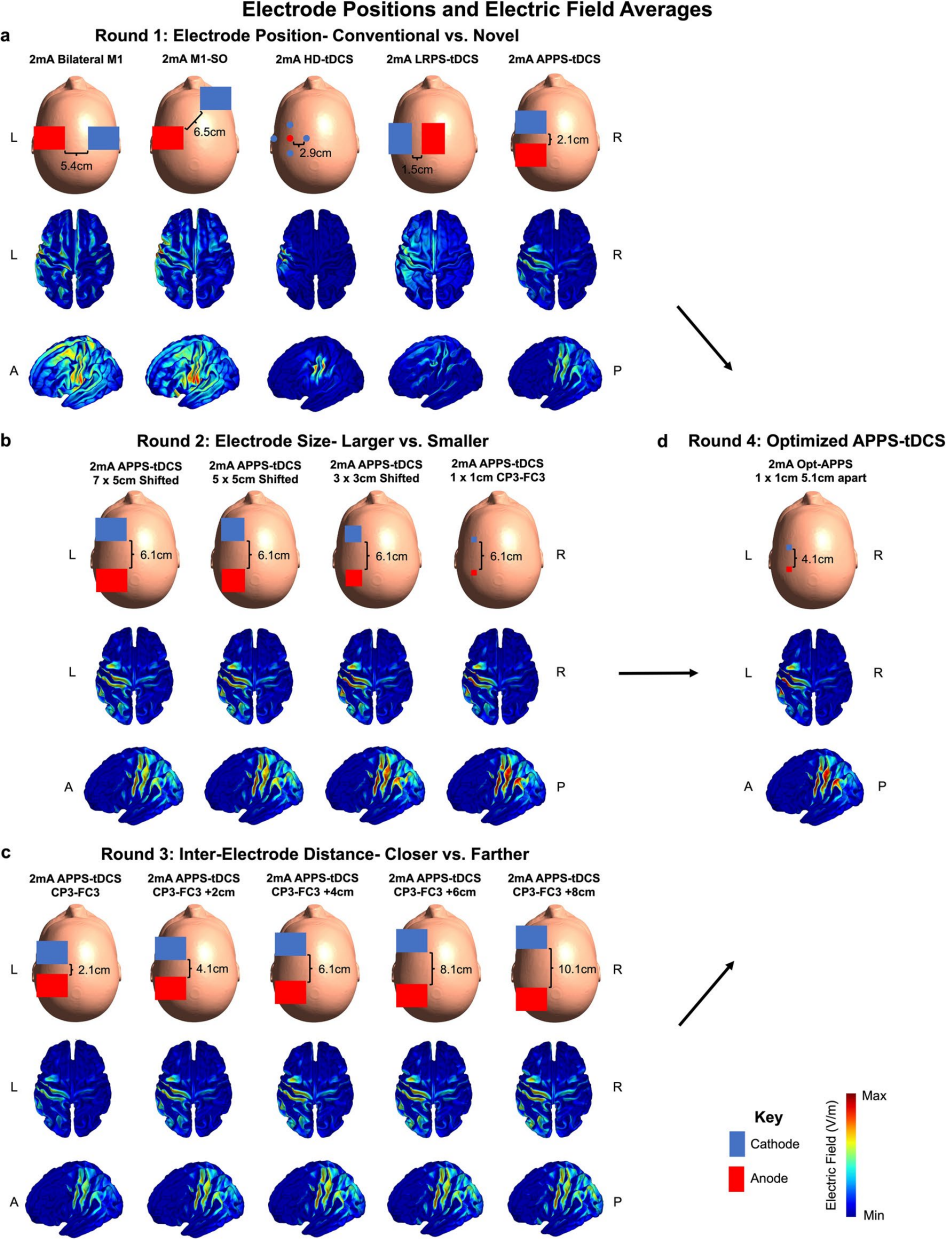
Electrode Montages and Visual Results of Electric Field Modeling. The top row of this figure shows the electrode positioning, sizes, and inter-electrode distances in Rounds 1–4 of modeling. For clarity, we show the electrode sizes on an MNI-152 head with relative electrode positioning as opposed to an actual head model used in the experiment. In addition, we show a top-down view and left hemispheric view of all 200 E-field models for each electrode set-up projected into fsaverage space. (**a**) Round 1 E-field modeling compared conventional bilateral M1, M1-SO, and HD-tDCS to novel left right pad surround (LRPS)-tDCS and anterior posterior pad surround (APPS)-tDCS electrode montages. APPS-tDCS produced the highest E-field magnitude, and the spread of stimulation was significantly more focal than in other pad electrode montages (i.e., bilateral M1 and M1-SO). (**b**) Round 2 E-field modeling kept the inter-electrode distance constant while varying the electrode size from 7 × 5 cm down to 1 × 1 cm, with an average of 6.12 cm between electrodes. Smaller 1 × 1 cm electrodes produced significantly higher and more focal E-fields than larger electrodes did. (**c**) Round 3 E-field modeling measured the effects of inter-electrode distance while keeping the electrode size constant at 7 × 5 cm. There was a non-linear increase in E-field magnitude, with the original APPS-tDCS position + 2 cm and + 4 cm producing significantly higher E-fields than other conditions. (**d**) Round 4 E-field modeling synthesized the results of electrode positioning, size, and inter-electrode distance with the goal of optimizing the stimulation parameters. This optimized APPS-tDCS montage used 1 × 1 cm electrodes positioned an average of 4.12 cm apart (i.e., placing the 1 × 1 cm electrodes at CP3-FC3 positions and moving them 2 cm closer together). Optimized APPS-tDCS induced significantly higher and more focal E-fields than the conventional electrode montages, producing the equivalent of 4.08 mA-like E-fields with only 2 mA of current.

Round 1 modeling compared five electrode montages (See Fig. 2 and Table 2 for full details): 1. 7 × 5 cm pad electrodes at bilateral C3 (anode) and C4 (cathode), with an average inter-electrode distance of 5.39 cm apart. 2. 7 × 5 cm pad electrodes at C3 (M1; anode) and right supraorbital (SO; cathode), with an average inter-electrode distance of 6.51 cm apart. 3. 0.5 cm diameter circular HD-tDCS (anode: C3 and cathodes: FC3, C1, CP3, C5), with average inter-electrode distances between the anode and four cathodal electrodes of 2.93 cm apart. 4. 7 × 5 cm Left Right Pad Surround (LRPS)-tDCS (anode: C1 and cathode: C5), with an average inter-electrode distance of 1.53 cm apart. 5. Anterior Posterior Pad Surround (APPS)-tDCS (anode: CP3, cathode: FC3), with an average inter-electrode distance of 2.12 cm apart. We visually ensured that electrodes did not touch or overlap for any of the 3000 models. Round 2 modeling systematically tested the effects of electrode size using APPS-tDCS. The four models compared 1 × 1 cm pad electrodes placed at anode: CP3, cathode: FC3, and 3 × 3 cm, 5 × 5 cm, and 7 × 5 cm pad electrodes that were individually adjusted to match the inter-electrode distance with the 1 × 1 cm electrodes, which were an average of 6.12 cm apart from the EEG coordinates. By keeping the electrode distance constant, we isolated the effects of electrode size. In Round 3 modeling, we used APPS-tDCS positioning and systematically isolated the variable of inter-electrode distance by keeping the electrode size constant at 7 × 5 cm pad electrodes. Here, we started with 7 × 5 cm pad electrodes centered at the original CP3 (anode)-FC3 (cathode) locations and sequentially moved the electrodes 1 cm farther anterior and posterior up to + 8 cm apart: 1. CP3-FC3; 2. CP3-FC3 + 2 cm; 3. CP3-FC3 + 4 cm; 4: CP3-FC3 + 6 cm. 5: CP3-FC3 + 8 cm. We moved these distances by starting with CP3-FC3 electrode coordinates and changing the anterior/posterior direction values to sequentially move 1 cm in the anterior and 1 cm in the posterior directions farther apart, as SimNIBS automatically finds the closest skin surface point^55^. Lastly, in Round 4 modeling, we took the optimal electrode position, size, and distance that maximized E-field magnitude highest on-target effects) while stimulating focally (fewest off-target effects) and computed a final optimized APPS-tDCS montage, with 1 × 1 cm electrodes placed at CP3-FC3 + 2 cm (average inter-electrode distance = 4.12 cm).

**Table 2:** Means, Standard Deviations, and Ranges of E-Field Magnitudes and Focality in Rounds 1–4 Modeling.

Round 1 Modeling				Round 2 Modeling				Round 3 Modeling					Round 4 Modeling
M1-SO	HD-tDCS	LRPS-tDCS	APPS-tDCS	APPS 7 × 5	APPS 5 × 5	APPS 3 × 3	APPS 1 × 1	APPS-tDCS	APPS + 2 cm	APPS + 4 cm	APPS + 6 cm	APPS + 8 cm	Opt-APPS
2 mA	2 mA	2 mA	2 mA	2 mA	2 mA	2 mA	2 mA	2 mA	2 mA	2 mA	2 mA	2 mA	2 mA
C3-Fp2	C3 Center	C1-C5	CP3-FC3	. + 4 cm	. + 4 cm	. + 2 cm	CP3-FC3	CP3-FC3	. + 2 cm	. + 4 cm	. + 6 cm	. + 8 cm	4.12 cm Apart
7 × 5 cm	0.5 cm diameter	7 × 5 cm	7 × 5 cm	7 × 5 cm	5 × 5 cm	3 × 3 cm	1 × 1 cm	7 × 5 cm	7 × 5 cm	7 × 5 cm	7 × 5 cm	7 × 5 cm	1 × 1 cm
0.273	0.306	0.326	0.363	0.428	0.462	0.509	0.549	0.363	0.421	0.428	0.418	0.403	0.553
0.053	0.126	0.067	0.081	0.077	0.087	0.108	0.133	0.081	0.084	0.077	0.071	0.065	0.138
0.145–0.469	0.076–0.098	0.178–0.574	0.185–0.784	0.214–0.537	0.224–0.829	0.231–1.009	0.235–1.238	0.185–0.784	0.207–0.774	0.214–0.751	0.215–0.732	0.214–0.691	0.228–1.151
0.392	0.209	0.297	0.316	0.412	0.434	0.47	0.489	0.316	0.386	0.412	0.421	0.423	0.47
0.061	0.086	0.056	0.069	0.074	0.082	0.107	0.135	0.069	0.076	0.074	0.073	0.07	0.126
0.276–0.769	0.089–0.734	0.169–0.568	0.181–0.77	0.243–0.783	0.25–0.857	0.25–1.09	0.237–1.41	0.181–0.77	0.221–0.756	0.243–0.783	0.254–0.811	0.264–0.795	0.22–1.15
1.51E + 05	1.07E + 04	4.21E + 04	2.75E + 04	5.71E + 04	5.37E + 04	4.04E + 04	2.66E + 04	2.75E + 04	3.95E + 04	5.71E + 04	7.87E + 04	1.01E + 05	2.14E + 04
3.42E + 04	2.93E + 03	9.26E + 03	4.98E + 03	9.43E + 03	9.39E + 03	6.65E + 03	4.68E + 03	4.98E + 03	6.72E + 03	9.43E + 03	1.33E + 03	1.76E + 03	3.73E + 03
4.98E + 04–2.55E + 05	4.77E + 03–1.99E + 04	1.95E + 04–7.4E + 04	1.59E + 04–4.31E + 04	3.52E + 04–8.27E + 04	3.22E + 04–8.26E + 04	2.45E + 04–5.77E + 04	1.31E + 04–3.74E + 04	1.59E + 04–4.31E + 04	2.42E + 04–6.0E + 04	3.52E + 04–8.27E + 04	4.74E + 04–1.22E + 05	5.75E + 04–1.65E + 05	1.17E + 04–3.17E + 04

### Outcome measures

**Figure 3:**
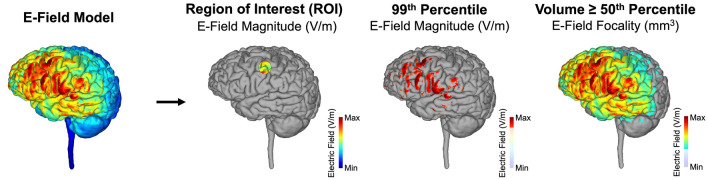
Primary Outcome Measures. For each E-field model, we performed three types of analyses: (1) Region of Interest (ROI) E-Field Magnitude. This method examines the average E-field magnitude in a 10 mm radius spherical ROI that was individually placed at the first gray matter voxel underneath the cortical projection at C3. This ROI was the same for every model and represented the E-field at the on-target cortical motor target. (2) 99th Percentile E-Field Magnitude. This method determined the E-field magnitude at the whole brain level, examining the average E-field in the top 1% of activated voxels irrespective of whether the effects were on-target or off-target. (3) E-Field Focality. We calculated focality by measuring the volume of voxels (mm^3^) equal to or above the 50th percentile E-field. The greater the volume stimulated, the lower the focality of stimulation (i.e., less focal = higher off-target effects).

For each model, we computed three outcome measures (Fig. 3): (1) Region of interest (ROI) E-field magnitude. This ROI represented the on-target E-field directly at the cortical target. We individually placed a 10 mm radius spherical ROI in the primary motor cortex for each person. This ROI was centered on the first gray matter voxel directly underneath the center of the C3 EEG location and used a gray matter mask to extract the average E-field in the gray matter voxels contained in the ROI volume. The gray matter mask was extracted in the segmentation step of headreco. On average, the MNI XYZ coordinates for the center of the motor ROI were: −55.18, −10.98, 46.65. We ensured that the center of the ROI was placed on a gyral crown and not a sulcal depth for each ROI, to minimize the variability that could be introduced by inconsistent between-subject ROI placements. Notably, the ROI was kept constant within-subject across all models, so any differences in ROI E-field were due to electrode position, size, or inter-electrode distance. (2) 99th percentile E-field magnitude. This 99th percentile measure represented the whole brain average E-field magnitude of the top 1% of voxels irrespective of location, which does not necessarily align with the ROI-based E-field. If the whole brain, 99th percentile E-field magnitude were similar to that of the ROI E-field at the target, this would suggest that the electrode montage successfully focused the maximum stimulation at the target. In contrast, if the 99th percentile and ROI E-field magnitudes diverged, this would indicate that maximum stimulation was off-target. (3) E-field focality. A further method of assessing on-target vs. off-target effects is E-field focality. This focality measure was calculated by taking the volume (mm^3^) of electric fields equal to or greater than the 50th percentile E-field. Lower volume stimulated indicated more focal stimulation, maximizing the on-target effects while limiting the off-target effects. In each model, we extracted the E-field magnitude (normE) and focality of E-field.

### Statistical analyses

We set the significance threshold at *p* < 0.05 for all statistical analyses (two-tailed). Rounds 1–4 of modeling were analyzed using repeated measures ANOVAs for each outcome measure in GraphPad PRISM 9.0.1. The E-fields produced in the optimized APPS-tDCS montage in Round 4 modeling were compared to the E-fields from the initial Round 1 models. All post-hoc analyses were Tukey corrected with significance level set at *p* < 0.05.

## Results

### Round 1: Effects of electrode positioning in conventional vs. novel electrode montages

**Figure 4:**
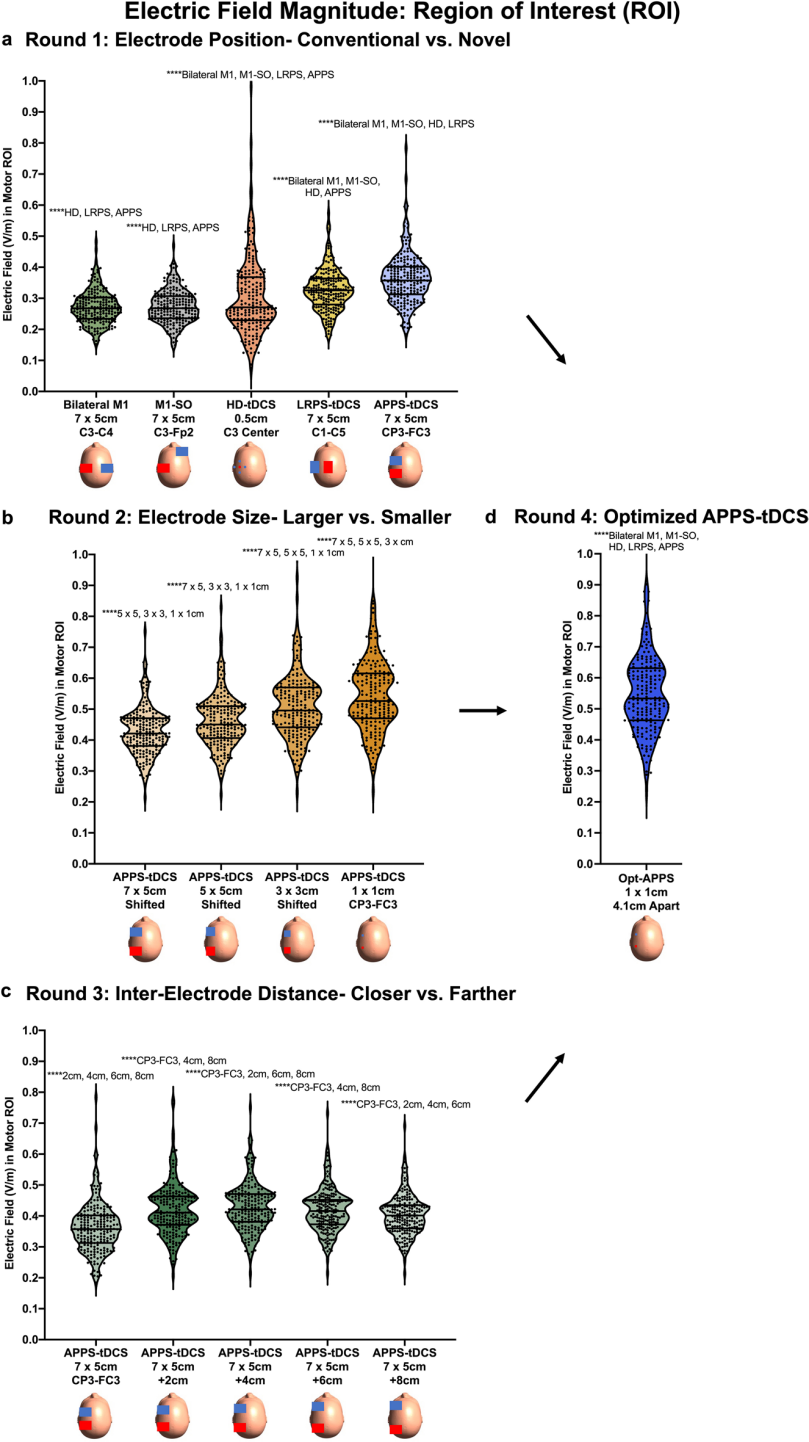
Electric Field Magnitude Results: Region of Interest (ROI) Analysis. This figure shows the E-field magnitudes at the motor ROI. (**a**) Round 1 E-field modeling demonstrated that placing electrodes surrounding the cortical target in anterior posterior pad surround (APPS)-tDCS produced significantly higher E-fields of 0.363 V/m (SD = 0.081 V/m), compared to 0.271 and 0.273 V/m in the bilateral M1 and M1-SO montages respectively (both SD = 0.053 V/m). (**b**) Round 2 E-field modeling showed how the smaller 1 × 1 cm electrode size resulted in significantly higher E-fields of 0.549 V/m (SD = 0.133 V/m), compared to 0.428 V/m (SD = 0.077 V/m) for the 7 × 5 cm electrode. (**c**) Round 3 E-field modeling showed a non-linear increase in E-field magnitude with greater distance, with the CP3-FC3 + 2 cm (mean = 0.421 V/m, SD = 0.084 V/m) and + 4 cm (mean = 0.428 V/m, SD = 0.077 V/m) inter-electrode distances producing the significantly highest E-field magnitudes. d) Round 4 E-field modeling used optimized parameters of APPS-tDCS with 1 × 1 cm electrodes that were placed an average of 4.12 cm apart. This optimized APPS-tDCS paradigm produced the highest E-field magnitude of 0.553 V/m (SD = 0.138 V/m), which was significantly higher than the E-field magnitude in each of the Round 1 modeling montages. ****p < 0.0001 (Tukey corrected).

In Round 1, we compared conventional electrode montages and two novel placements surrounding the cortical target. In our ROI analysis, we found that varying electrode positioning produced significantly different electric fields at the motor ROI, F(1.8, 361.4) = 169.0, *p* < 0.0001. Post-hoc Tukey corrected analyses indicated that each comparison statistically differed at the *p* < 0.0001 significance level except for the conventional bilateral M1 and M1-SO montages (Fig. 4a). In particular, APPS-tDCS electrodes that surround the cortical motor target by placing electrodes in the anterior and posterior directions produced the highest mean E-field of 0.363 V/m (SD = 0.081 V/m), compared to LRPS-tDCS (mean = 0.326 V/m, SD = 0.067 V/m), HD-tDCS (mean = 0.306 V/m, SD = 0.126 V/m), bilateral M1 (mean = 0.271 V/m, SD = 0.054 V/m), and M1-SO (mean = 0.273 V/m, SD = 0.053 V/m) (Fig. 4a; Table 2).

**Figure 5:**
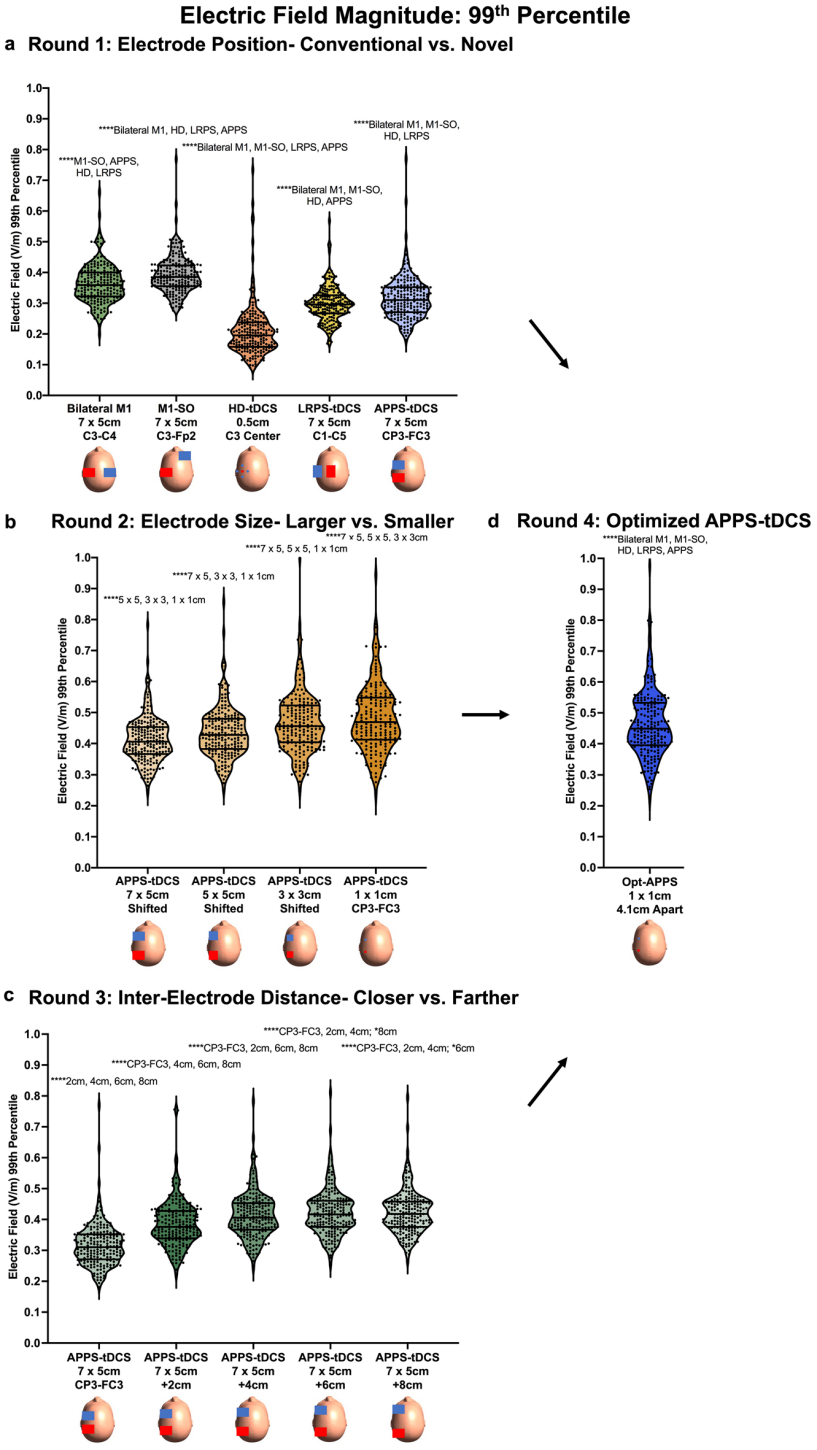
Electric Field Magnitude Results: 99th Percentile Analysis. In Fig. 5, we analyzed the E-field magnitude using the commonly utilized 99th percentile threshold approach. (**a**) Round 1 E-field modeling. In contrast to the ROI approach, bilateral M1 (mean = 0.363 V/m, SD = 0.063 V/m) and M1-SO (mean = 0.392 V/m, SD = 0.061 V/m) 99th percentile E-field magnitudes were significantly higher than those induced by APPS-tDCS (mean = 0.316 V/m, SD = 0.069 V/m). This disparity of the E-field magnitudes between the ROI and 99th percentile approaches suggests that the maximal E-field produced from conventional electrode montages is not underneath the electrodes as intended. Supplementary Sects. 1–2 further examined the location of maximal E-field magnitude, finding that it is midway between the electrodes and explaining why APPS-tDCS produces the highest ROI E-field at the intended motor target. (**b**) Round 2 E-field modeling showed that smaller 1 × 1 cm electrodes produced the highest 99th percentile E-field (mean = 0.489 V/m, mean = 0.135 V/m), compared to the larger 7 × 5 cm electrodes (mean = 0.412 V/m, SD = 0.074 V/m). (**c**) Round 3 E-field modeling showed that increasing the distance between electrodes produced higher 99th percentile E-field magnitudes but in a non-linear fashion (highest 99th percentile E-field: + 8 cm from the CP3-FC3 location). Thus, these data suggest that the researcher should weigh the benefit of increased E-field magnitude against lower focality, which is inherent to increasing the inter-electrode distances. (**d**) Round 4 E-field modeling with optimized parameters (APPS-tDCS with 1 × 1 cm electrodes placed an average of 4.12 cm apart) produced a 99th percentile E-field of 0.47 V/m (SD = 0.126 V/m), which was significantly higher than the 99th percentile E-fields in Round 1 modeling montages ****p < 0.0001 (Tukey corrected).

The 99th percentile E-field magnitude was also significantly impacted by electrode positioning, F(1.9, 371.9) = 843.6, *p* < 0.0001 (Fig. 5a and Table 2). Post-hoc Tukey corrected tests revealed that the conventional bilateral M1 and M1-SO montages produced the two highest 99th percentile E-fields of 0.363 V/m (SD = 0.063 V/m) and 0.392 V/m (SD = 0.061 V/m) respectively. These values were significantly higher than HD-tDCS (mean = 0.209 V/m, SD = 0.086 V/m), LRPS-tDCS (mean = 0.297 V/m, SD = 0.056 V/m), and APPS-tDCS (mean = 0.316 V/m, SD = 0.069 V/m). Each post-hoc comparison was significant at the *p* < 0.0001 significance level.

**Figure 6:**
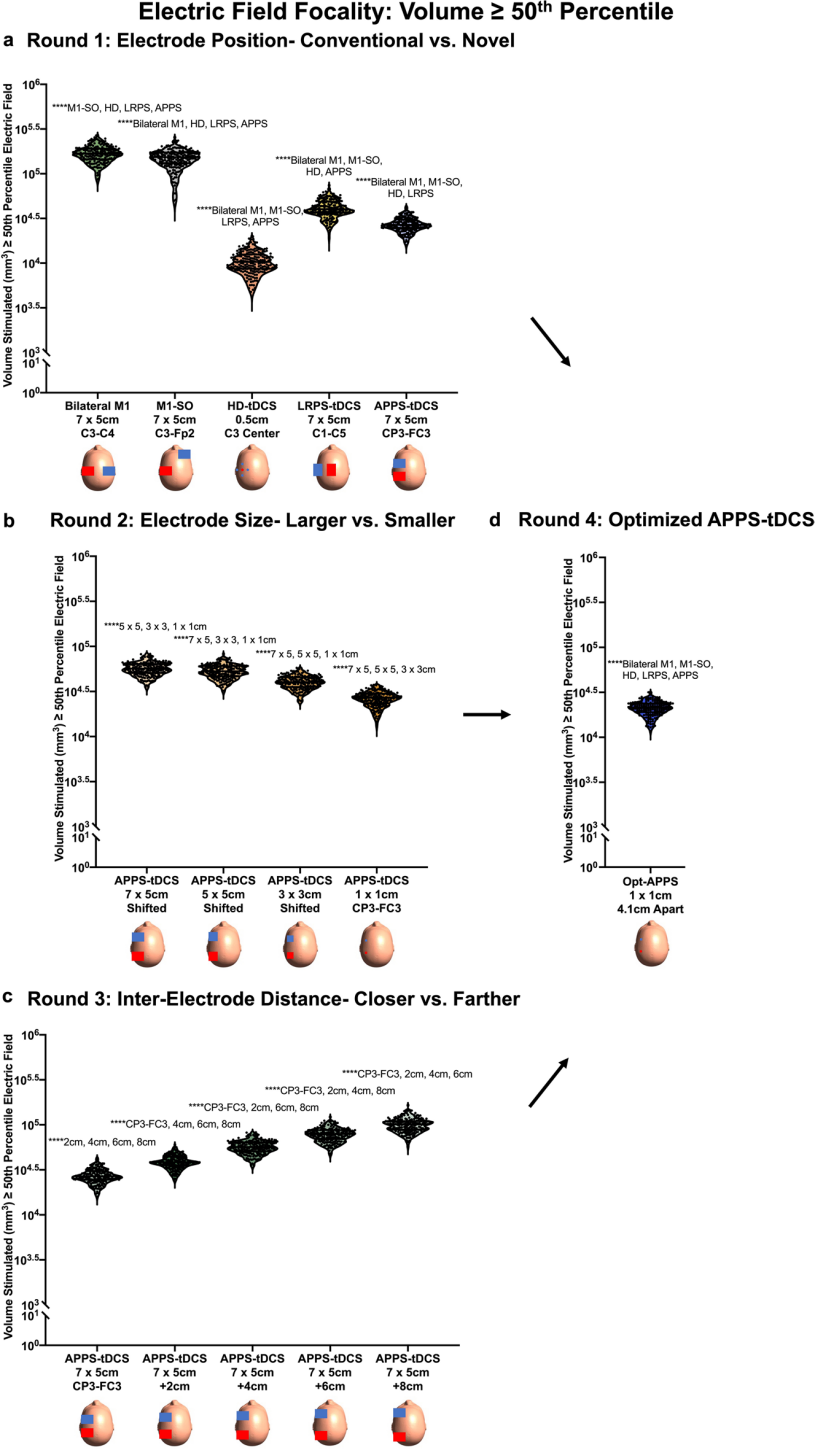
Electric Field Focality Results. In this figure, we analyzed the focality of stimulation as a function of the volume stimulated (mm^3^) that was greater than or equal to the 50th percentile E-field. A higher volume stimulated represented less focal stimulation whereas lower volume stimulated represents more focal, on-target stimulation. Subfigures a-d represent Rounds 1–4 of E-field modeling respectively. Across all rounds of modeling, HD-tDCS was the most focal type of tDCS, stimulating a volume of only 1.07 × 10^4^ mm^3^ (SD = 2.93 × 10^3^ mm^3^). The second most focal stimulation type was optimized APPS-tDCS in Round 4 modeling, stimulating a volume of 2.14 × 10^4^ mm^3^ (SD = 3.73 × 10^3^ mm^3^). Round 2 modeling demonstrated that the smaller 1 × 1 cm electrodes stimulated the most focal volume, and Round 3 modeling showed that the volume stimulated linearly increased with the inter-electrode distance. See Table 2 for details. ****p < 0.0001 (Tukey corrected).

Regarding focality, electrode placement significantly altered the spread of electric fields, F(2.0, 402.8) = 2975, *p* < 0.0001 (Fig. 6a and Table 2). Tukey corrected post-hoc comparisons revealed that the volume stimulated from each electrode montage significantly differed at the *p* < 0.0001 significance level. HD-tDCS delivered the most focal amount of stimulation at 1.07 × 10^4^ mm^3^ (SD = 2.93 × 10^3^ mm^3^). Of the pad electrode montages, APPS-tDCS delivered the most focal amount of stimulation (mean = 2.75 × 10^4^ mm^3^, SD = 4.98 × 10^3^ mm^3^), followed by LRPS-tDCS (mean = 4.21 × 10^4^ mm^3^, SD = 9.26 × 10^3^ mm^3^), M1-SO (mean = 1.51 × 10^5^ mm^3^, SD = 3.42 × 10^4^ mm^3^), and bilateral M1 (mean = 1.70 × 10^5^ mm^3^, SD = 3.42 × 10^4^ mm^3^).

In sum, APPS-tDCS produced the highest E-field magnitude at the motor ROI by placing standard 7 × 5 cm electrodes surrounding the cortical motor target in the anterior (FC3) and posterior (CP3) directions (i.e., highest on-target effects). In Supplementary Sect. 1, we used a further analysis to confirm that APPS-tDCS consistently produced a maximal E-field midway between the electrodes in all 200 participants. A subsequent analysis in Supplementary Sect. 2 found higher intraclass correlation coefficient (ICC) scores between ROI and 99th percentile E-field magnitudes for APPS-tDCS compared to all other electrode montages, which is indicative of the electrodes surrounding the target producing the most focused and highest E-field magnitude at the intended target. However, since we found that the conventional bilateral M1 and M1-SO pad electrode montages produced higher whole brain, 99th percentile E-fields than all other conditions, there is still room for further optimization. Likewise, HD-tDCS produced the most focal stimulation, with even the more focal APPS-tDCS approach stimulating over double the volume. Further optimizing APPS-tDCS, by systematically testing the effects of electrode size and inter-electrode distance, was the focus of Rounds 2–3 modeling.

### Round 2: Effect of electrode size in APPS-tDCS

In Round 2 of modeling, we compared the effects of electrode size on the ROI, 99th percentile, and focality of E-fields using 1 × 1 cm, 3 × 3 cm, 5 × 5 cm, and 7 × 5 cm electrodes with matched inter-electrode distances. For the motor ROI, the E-field magnitude significantly differed by electrode size, F(1.1, 209.9) = 730.4, *p* < 0.0001 (Fig. 4b; Table 2). Post-hoc analyses revealed that the smallest 1 × 1 cm electrode size had the highest E-field at the motor ROI (mean = 0.549 V/m, SD = 0.133 V/m), compared to 3 × 3 cm (mean = 0.509 V/m, SD = 0.108 V/m), 5 × 5 cm (mean = 0.462 V/m, SD = 0.087 V/m), and 7 × 5 cm (mean = 0.428 V/m, SD = 0.077 V/m). All post-hoc comparisons were significant at the Tukey-corrected *p* < 0.0001 level.

Using the 99th percentile threshold, there was also a significant difference between the E-field magnitude produced by varying electrode sizes, F(1.0, 208.7) = 236.5, *p* < 0.0001, Fig. 5b and Table 2). Similar to the ROI analysis,1 × 1 cm electrodes produced the highest whole brain peak E-field of 0.489 V/m (SD = 0.135 V/m), followed by the 3 × 3 cm (mean = 0.47 V/m, SD = 0.107 V/m), 5 × 5 cm (mean = 0.434 V/m, SD = 0.082 V/m), and 7 × 5 cm electrodes (mean = 0.412 V/m, SD = 0.074 V/m).

Lastly, focality inversely scaled as a function of electrode size, such that the smallest electrodes produced the most focal E-fields, F(1.3, 251.4) = 3398, *p* < 0.0001 (Fig. 6b; Table 2). The 1 × 1 cm electrodes stimulated the smallest volume ≥ 50th percentile E-field of 2.66 × 10^4^ mm^3^ (SD = 4.68 × 10^3^ mm^3^), compared to 3 × 3 cm (mean = 4.04 × 10^4^ mm^3^, SD = 6.65 × 10^3^ mm^3^), 5 × 5 cm (mean = 5.37 × 10^4^ mm^3^, SD = 9.39 × 10^3^ mm^3^), and 7 × 5 cm electrodes (mean = 5.71 × 10^4^ mm^3^, SD = 9.43 × 10^3^ mm^3^).

Summarizing the Round 2 modeling results, electrode size significantly affects the E-field magnitude at the ROI and 99th percentile resolutions, as well as the focality of stimulation. When keeping inter-electrode distance constant to isolate the impact of electrode size, 1 × 1 cm electrodes produce the highest and most focal E-fields (i.e., highest on-target and lowest off-target effects). Further optimizing inter-electrode distance based on set distance variations from 10 to 10 EEG coordinates was the focus of Round 3 modeling.

### Round 3: Effect of inter-electrode distance in APPS-tDCS

Round 3 of modeling kept electrode size constant at the 7 × 5 cm size and moved electrodes farther apart by + 2 cm increments, up to + 8 cm, to evaluate the impact of electrode distance on electric field magnitude and focality. This altered the edge-to-edge distance between electrodes from an average of 2.12 cm apart at the original CP3-FC3 positioning, up to 10.12 cm apart (Fig. 2c).

Electrode distance significantly affected E-field magnitude in the motor ROI, F(1.4, 274) = 502.8, *p* < 0.0001. There was a significant increase in E-field magnitude from the initial APPS-tDCS model with 7 × 5 cm electrodes placed at the 10–10 EEG locations of CP3-FC3 (mean = 0.363 V/m, SD = 0.081 V/m) to + 2 cm (mean = 0.421 V/m, SD = 0.077 V/m) and + 4 cm (mean = 0.428 V/m, SD = 0.084 V/m). At the + 6 cm (mean = 0.418 V/m, SD = 0.071 V/m), and + 8 cm (mean = 0.403 V/m, SD = 0.065 V/m), there were higher E-field magnitudes than the initial CP3-FC3 APPS-tDCS locations (Fig. 4c). However, there were diminishing returns of E-field magnitude gains from moving the electrodes farther apart, indicating that inter-electrode distance has a non-linear relationship with E-field magnitude and that the + 2 cm or + 4 cm distance (average inter-electrode distance of 4.12 cm or 6.12 cm apart) may be optimal to maximize the on-target E-field magnitude while minimizing the off-target effects from increasing the inter-electrode distance. While beyond the scope of this project, it is likely that this non-linearity from increasing the electrode distance is due to the dispersion of the electric field through a larger volume of brain tissue and focal point of the maximal electric field centered farther down in subcortical areas.

Regarding the 99th percentile outcome measure, there was a significant effect of distance on E-field magnitude, F(1.4, 273.7) = 2065, *p* < 0.0001 (Fig. 5c; Table 2). Like the ROI E-field magnitude, there was a non-linear increase of E-field from the initial model to + 8 cm: CP3-FC3 (mean = 0.316 V/m, SD = 0.069 V/m) to + 2 cm (mean = 0.386 V/m, SD = 0.076 V/m), + 4 cm (mean = 0.412 V/m, SD = 0.074 V/m), + 6 cm (mean = 0.421 V/m. SD = 0.073 V/m), and + 8 cm (mean = 0.423 V/m, SD = 0.07 V/m). Whole brain E-field magnitudes appear to have maximal gains from the initial distance increases from the initial APPS-tDCS positions of CP3-FC3 and a plateauing effects as electrodes are moved farther apart.

Finally, the inter-electrode distance significantly and linearly affected the amount of volume stimulated, F(1.2, 243.1) = 3821, *p* < 0.0001 (Fig. 6c and Table 2). The baseline CP3-FC3 APPS-tDCS positioning stimulated a volume of 2.75 × 10^4^ mm^3^ (SD = 4.98 × 10^3^ mm^3^), + 2 cm stimulated 3.96 × 10^4^ mm^3^ (SD = 6.72 × 10^3^ mm^3^), + 4 cm stimulated 5.71 × 10^4^ mm^3^ (SD = 9.43 × 10^3^ mm^3^), + 6 cm stimulated 7.87 × 10^4^ mm^3^ (SD = 1.33 × 10^4^ mm^3^), and + 8 cm stimulated 1.01 × 10^5^ mm^3^ (SD = 1.76 × 10^4^ mm^3^).

In sum, Round 3 modeling demonstrated that inter-electrode distance significantly affects E-field magnitude and focality of stimulation. However, E-field magnitude at the motor ROI increased from the initial APPS-tDCS position to + 2 cm and + 4 cm, with lower E-fields from further increasing the inter-electrode distance. While the whole brain 99th percentile E-fields increased with each subsequent distance, the magnitude plateaued with diminishing gains from moving electrodes farther apart. Thus, when considering inter-electrode distance for tDCS montages, it is important to weigh not only the consideration of maximal on-target E-fields but also the volume of off-target effects. In Round 4 modeling, we aimed to optimize the APPS-tDCS parameters that would maximize the on-target E-field magnitude while minimizing the off-target effects.

### Round 4: Optimized APPS-tDCS

In a final electrode montage, we optimized tDCS parameters by combining the best electrode placement (APPS-tDCS), size (1 × 1 cm), and distance (moving 2 cm closer together than the CP3-FC3 coordinates with 1 cm electrodes, for an average of 4.12 cm apart). We statistically compared these optimized APPS-tDCS results with the original five models to compare the effects to the bilateral M1, M1-SO, HD-tDCS, LRPS-tDCS, and original 7 × 5 cm APPS-tDCS montages. Repeated measures ANOVAs were significant for ROI (F(1.7, 348) = 977.2, *p* < 0.0001), 99th percentile E-field (F(2.6, 512.9) = 898.2, *p* < 0.0001, and focality (F(2.0, 395.2) = 3079, *p* < 0.0001 (Figs. 4d, 5d, and 6d; Table 2).

Post-hoc Tukey corrected analyses (all *p* < 0.0001) showed that this optimized APPS-tDCS placement produced the highest motor ROI E-field magnitude of 0.553 V/m (SD = 0.138 V/m) and significantly higher 99th percentile E-field of 0.47 V/m (SD = 0.126 V/m) compared to each Round 1 modeling montage. In addition, optimized APPS-tDCS stimulated the significantly lowest volume out of the conventional pad electrode montages, with 2.14 × 10^4^ mm^3^ stimulated (SD = 3.73 × 10^3^ mm^3^). Taken together, optimized APPS-tDCS appears to produce high on-target and relatively low off-target effects.

## Discussion

In this study, we used large scale E-field modeling to systematically test the effects of electrode positioning, size, and distance on E-field magnitude and focality. Our goal was to derive a new form of more efficient tDCS that maximizes the on-target E-field while minimizing the off-target effects. Similar to how Datta et al. (2009) used computational modeling to develop 4 × 1 HD-tDCS prior to human application^43^, here we provided a theoretical basis for how to optimize tDCS electrode positioning based on simple 10–10 EEG coordinates in four rounds of E-field modeling. Round 1 modeling demonstrated that placing tDCS electrodes surrounding the motor target in the anterior and posterior directions (i.e., APPS-tDCS) produced a 33.9% higher E-field from the same 2 mA stimulation intensity while stimulating only 16.2% of the volume compared to conventional bilateral M1 and M1-SO montages (i.e., higher on-target, fewer off-target effects). Working with this APPS-tDCS montage, in Round 2 modeling we found that smaller 1 × 1 cm electrodes produced higher and more focal E-fields than 3 × 3 cm, 5 × 5 cm, and 7 × 5 cm electrodes with the same inter-electrode distance, up to an average of 202.5% higher on-target stimulation with only 15.6% of the volume stimulated with 1 × 1 cm APPS-tDCS electrodes placed at CP3-FC3. Furthermore, Round 3 modeling demonstrated the importance of inter-electrode distance, with non-linear increases of E-field magnitude from electrodes placed increasingly farther apart. Synthesizing these concepts in Round 4 modeling, we derived an optimized APPS-tDCS montage, surrounding the cortical motor target with 1 × 1 cm electrodes placed + 2 cm closer together than the CP3-FC3 10–10 EEG locations (i.e., average of 4.12 cm apart). This optimized APPS-tDCS electrode positioning produced over double (204.1%) the on-target E-field as conventional bilateral M1 and M1-SO montages while stimulating a fraction (12.6%) of the off-target cortical areas. Put in other terms, optimized 2 mA APPS-tDCS produces the equivalent on-target E-field magnitude as 4.08 mA tDCS using conventional bilateral M1 or M1-SO montages with 7 × 5 cm electrodes.

There are many implications from these findings. First, this study provides a theoretical basis for optimized tDCS that maximizes the on-target effects while minimizing the off-target effects. All 2 mA stimulation is not the same. Rather, 2 mA that is strategically applied can stimulate the cortex at significantly different intensities (Fig. 2). Given the emerging interest in applying tDCS at higher scalp intensities^29,30,35,36^, perhaps an alternative strategy could be to utilize the principles outlined in this paper to deliver more efficient stimulation at 2 mA intensities. Namely, more efficient 2 mA APPS-tDCS could mitigate the potential increase in adverse effects and lower tolerability that may be inherent to higher intensity stimulation (e.g., more scalp burning or irritation from higher 4 mA tDCS intensities). Alternatively, researchers could utilize optimized APPS-tDCS to deliver the same levels of cortical stimulation with lower scalp intensities; 2 mA-like effects from conventional bilateral M1 or M1-SO montages could be achieved with 0.98 mA stimulation with optimized APPS-tDCS. Prospective safety and tolerability testing of APPS-tDCS at various intensities is warranted.

A second goal of this study was to derive a new, efficient tDCS stimulation strategy based on simple EEG coordinates. Since tDCS is uniquely appealing for its ease of use and ability to be self-administered in an at-home environment^18–22^, it is important to consider how easy it would be to implement new tDCS electrode positioning strategies. Taking this into consideration, we sought to develop a straightforward, easily replicable tDCS approach relying on 10–10 EEG coordinates as opposed to MRI-based individualized dosing or electrode positioning. APPS-tDCS places electrodes based at the CP3 (anode) and FC3 (cathode) locations, which are easily identifiable with scalp measurements. Centering 1 × 1 cm electrodes at these positions in Round 2 modeling resulted in E-field magnitudes of 202.5% the intensity of conventional bilateral M1 or M1-SO montages with only 15.6% of the volume stimulated (i.e., higher on-target and fewer off-target effects; Fig. 2b). Optimizing distance on top of the 1 × 1 cm electrodes by distancing the electrodes an average of 4.12 cm apart resulted in marginally higher E-field magnitude (204.1% conventional) and greater focality (12.6% volume stimulated). The focality of stimulation was not quite as high as in HD-tDCS (6.3% of the volume stimulated as conventional M1-SO electrode placements), but was the most focal stimulation of any pad electrode stimulation method. Taken together, we demonstrated how surrounding the cortical target with tDCS electrodes can result in higher and more focal E-fields at the same stimulation intensity and without the typically observed inverse relationship between E-field magnitude and focality^50,51^. Since the most optimized approach we presented in Round 4 modeling relies on slightly more complex measurements (i.e., 1 cm adjustments) on top of 10–10 EEG measurements, we welcome researchers to consider which electrode positioning, size, and inter-electrode distance works best for their needs. To this point, Round 2 modeling that centered 1 × 1 cm electrodes on the CP3-FC3 coordinates already produced more than double the on-target E-fields as conventional montages without these additional measurements and might be preferable for easily implemented at-home applications.

Third, these data highlight the importance of considering electrode positioning when examining prior tDCS results. Specifically, it is possible that previous tDCS results are varied in part due to the heterogeneity of stimulation protocols. Our data show that the same stimulation intensity of 2 mA can result in widely varying amounts of stimulation reaching the cortex depending on the electrode positioning, sizes, and inter-electrode distances. Considering that prior retrospective E-field analyses have found that higher E-field magnitudes are associated with stronger behavioral outcomes^33,34^, perhaps the prior studies finding stronger results utilized more optimal tDCS electrode positioning producing higher on-target E-fields. A recent meta-analysis considered the effects of electrode position and stimulation intensity on E-field magnitudes and subsequent working memory improvements, finding that more lateral prefrontal stimulation results in stronger behavioral effects^57^. Continuing to consider the effects of electrode positioning, size, and distance in prior and future studies could be informative and help to improve the behavioral effects induced by tDCS.

Finally, the results we presented in this study provide a starting point for considering the E-field averages and ranges from conventional bilateral M1, M1-SO, and 4 × 1 HD-tDCS. When considering reverse-calculation E-field dosing^32,37^ or computational approaches for determining an optimal electrode montage for maximal E-fields at the cortical target^38^, the user still must choose the cortical E-field threshold to base the scalp dosage at. It is currently unclear what the optimal E-field threshold is, and whether this would differ depending on the outcome measure (i.e., ROI vs. 99th percentile). Furthermore, it is unknown whether there is a linear dose–response relationship between E-field magnitude or if it is simply important to be above a certain threshold. This study presents the E-field thresholds from 2 mA stimulation in a large, paired dataset of 3000 E-field models in 200 healthy adults, providing a starting point for individualized E-field dosing. For instance, researchers could use these data to base individualized E-field thresholds at, above, or below the group-level E-field averages produced from 2 mA in various montages. Notably, the optimized APPS-tDCS montage we presented here interfaces both with common tDCS devices and individualized E-field modeling, making the future applications of this technique easily and widely adopted.

### Limitations and future directions

There are several limitations and future directions of this study. Electric field modeling is inherently limited by its use of MRI scans to estimate, but not directly measure, how much stimulation reaches the cortex. Future studies should prospectively test the APPS-tDCS approach to determine the tolerability of smaller electrode stimulation, ease of application, and behavioral effects, which we hypothesize to be greater than that of conventional 2 mA stimulation. Important foundational research comparing E-field modeling estimates and actual intracranial recordings established a strong linear relationship between these values, mitigating some but not all of the concern that E-field modeling is an estimation of cortical stimulation^58,59^. Further improvements in E-field modeling methodology, such as taking different sub-layers of meninges^60^, skull composition^61^, or anisotropy^62^ into consideration, may help to further refine the fidelity of E-field models. A further limitation is that we combined relative (i.e., 10–10 EEG-based electrode placements) and absolute measurements (i.e., 2 cm increments further apart), which resulted in slight disparities of up to a few millimeters between inter-electrode distances between participants. Furthermore, to systematically move electrodes farther apart, we only changed the anterior/posterior direction values and relied on the SimNIBS program automatically determining the closest skin surface. This may have led to slight deviations when compared to accounting for the scalp curvature. A future line of research could further elucidate how to best optimize electrode positioning based on inter-electrode distance with all values measured on a relative, subject-specific scale and accounting for scalp curvature.

While we examined E-field magnitude and focality in this study, it may also be informative to also examine the E-field direction for the stimulation outcome, as has been examined in prior studies^45^. Furthermore, adding diffusion imaging scans may help to further improve the accuracy of the E-field models and is the topic of ongoing research^63–65^. In addition, noninvasive brain stimulation often tests neurophysiology in the motor circuit, with the assumption that motor-induced effects are generalizable to other brain regions such as the prefrontal cortex^66,67^. Prior research simulating E-fields produced by transcranial magnetic stimulation (TMS) has demonstrated that there are differences in E-field magnitude in the motor vs. prefrontal cortices^68^, which appears to be due to varying skull thicknesses and scalp-to-cortex distances between frontal, parietal, temporal, and occipital areas^69^. Therefore, future directions of our research include modeling and prospectively applying APPS-tDCS over the prefrontal and parietal cortices to determine how these principles of electrode positioning, size, and inter-electrode distance in the motor cortex might apply to different brain regions. Finally, in this study we demonstrated how APPS-tDCS electrode positioning can increase the on-target E-field magnitude in the motor cortex, but what we did not address is whether there is an optimal E-field magnitude. Researchers have previously found retrospective dose–response relationships between E-field magnitude and clinical response in depression^33^ and working memory^34^, but further research in this area is needed to refine our understanding of optimal E-field magnitude.

### Conclusions

In summary, we performed 3000 electric field models in 200 HCP participants to test the effects of tDCS electrode position, size, and inter-electrode distance on E-field magnitude and focality of stimulation. Applying APPS-tDCS, with electrodes surrounding the cortical target in the anterior and posterior directions, delivers more than double the on-target electric fields and a fraction of the off-target effects from the same 2 mA stimulation intensity as conventional electrode placements. APPS-tDCS uses simple 10–10 EEG coordinates and can be easily adoptable using any existing, commercially available tDCS device. Prospective research using APPS-tDCS could ultimately result in stronger or more consistent transdiagnostic therapeutic effects and further testing is warranted.

## Supplementary Information

Supplementary Information.

## Data Availability

The MRI data are freely available from the open access Human Connectome Project database (http://www.humanconnectomeproject.org/data/).
