# Supplementary material for: Optimized APPS-tDCS electrode position, size, and distance doubles the on-target stimulation magnitude in 3000 electric field models
Source: Sci Rep. 2022 Nov 22;12:20116. doi: 10.1038/s41598-022-24618-3 (PMC9684449; doi:10.1038/s41598-022-24618-3)
Supplement: Supplementary file 1 — Supplementary Information. [file 41598_2022_24618_MOESM1_ESM.docx]

**APPS-tDCS Supplementary Sections 1-2**

To accompany: Caulfield & George ‘Optimized APPS-tDCS electrode position, size, and distance doubles the on-target stimulation magnitude in 3000 electric field models’

**Supplementary Section 1: Location of the Maximal Electric Field Underneath vs. Between APPS-tDCS Electrodes**

**Round 1** electric field (E-field) modeling visually showed that the maximal E-field is between and not underneath the electrodes since positioning the electrodes surrounding the cortical motor target produced higher E-fields in the motor region of interest (ROI). As a second method of investigating the location of the maximal E-field, and to confirm that this effect is not only observed at a group level but also at an individual level, we examined the E-field magnitude at three ROIs for the anterior posterior pad surround (APPS)-tDCS 7 x 5cm model (**Supplementary Figure 1a**).

We placed the three ROIs at the same motor cortical target underneath C3, and at individually placed cortical grey matter projections underneath the center of the anodal (CP3) and cathodal (FC3) electrodes using the same 10mm radius spherical ROI shape (**Supplementary Figure 1b**). Quantitatively (**Supplementary Figure 1c**) and visually (**Supplementary Figure 1d**), the group level maximal E-field was midway between electrodes at the motor ROI (mean = 0.363V/m, SD = 0.081V/m), as opposed to underneath the anode at 0.172V/m (SD = 0.043V/m) and cathode at 0.201V/m (SD = 0.052V/m). Furthermore, this effect was consistent between individuals, with each of the 200 participants having the maximal E-field midway between, and not underneath, the electrodes. Thus, the maximal E-field is repeatedly induced midway between the electrodes in each person, further affirming the utility in using APPS-tDCS to place electrodes surrounding the cortical target to maximize the E-field.

**Supplementary Figure 1:**

**
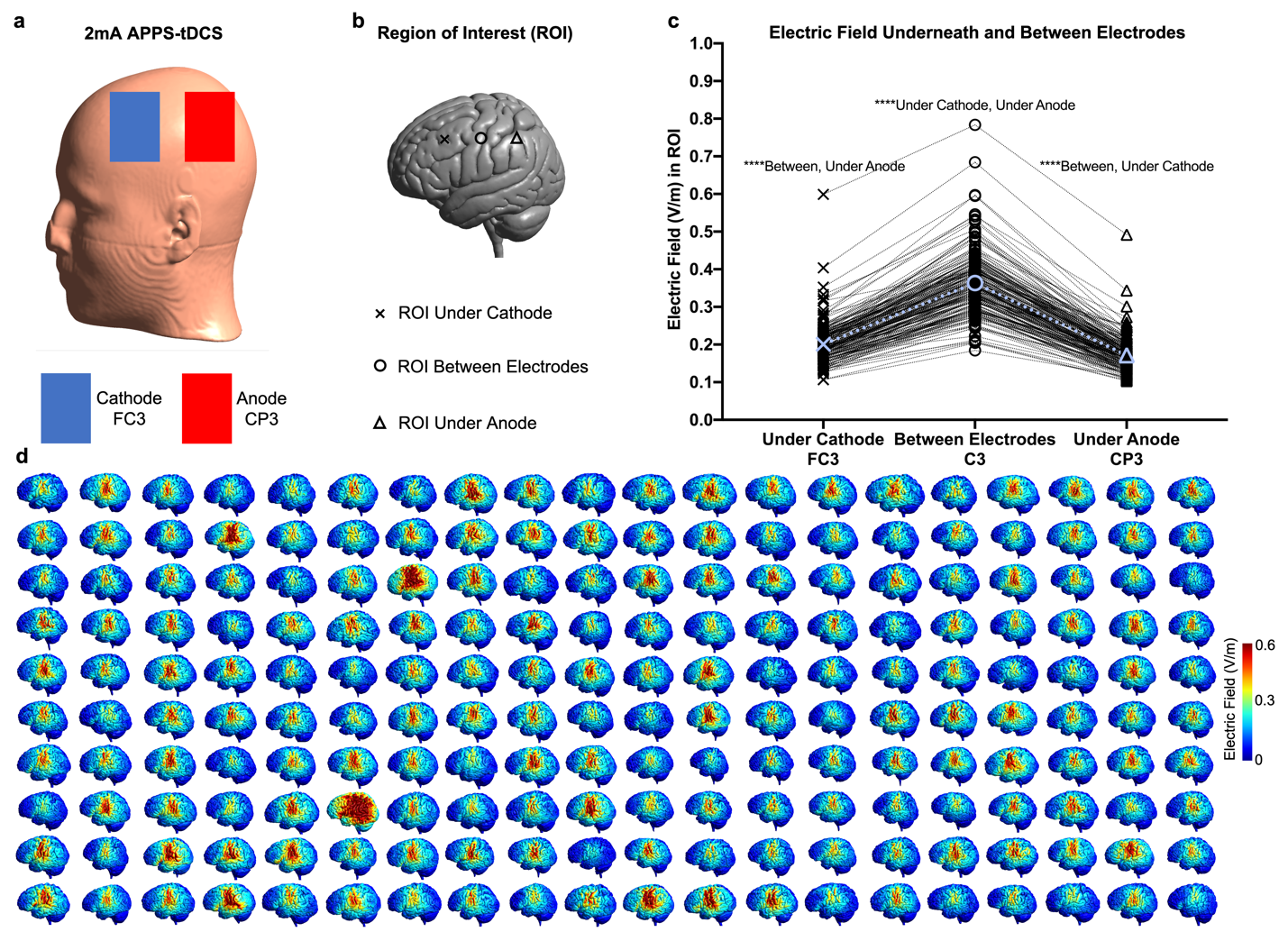
**

**Supplementary Figure 1: Region of Interest (ROI) Analyses Underneath vs. Between Electrodes in APPS-tDCS. a)** Location of APPS-tDCS anodal (CP3) and cathodal (FC3) electrodes. **b)** ROI Locations under the cathode (X), between electrodes (circle), or under the anode (triangle). **c)** Quantitative Evaluation of the E-field magnitude in each ROI. The maximum E-field for each of the 200 participants was midway between the electrodes (****p < 0.0001), affirming the APPS-tDCS strategy of surrounding the cortical target for maximizing induced E-fields. The E-field between each electrode (mean = 0.363V/m) was significantly greater than that of the E-fields underneath the anode (mean = 0.172V/m) and cathode (mean = 0.201V/m).

The blue symbols and dotted line denote the group average while the black symbols and lines show the paired data for each participant. **d)** Visualization of 200 E-field models using APPS-tDCS. Qualitatively, the appearance of the APPS-tDCS induced E-fields confirms that the maximal E-field is midway and not underneath the electrodes. These data call into question the electrode placement strategy of nearly every tDCS application to date, as electrodes are typically placed directly over the cortical target; APPS-tDCS may be a more efficient method of stimulation that focuses the maximal E-field on the cortical target by placing the electrodes surrounding, and not directly over, the intended stimulation area. Images were generated in SimNIBS 3.2.3 (<https://simnibs.github.io/simnibs/build/html/index.html>) and the graph was generated in GraphPad PRISM 9.0.1 (<https://www.graphpad.com/scientific-software/prism/>).

**Supplementary Section 2: Similarity Analyses Between Motor Region of Interest (ROI) and 99^th^ Percentile Whole Brain Peak Electric Fields**

A second method to assess whether a given electrode montage induces the peak electric field at the intended cortical target is to compare the similarity between the whole brain 99^th^ percentile and region specific ROI electric fields. If the E-field in the top 1% of voxels (extracted in the 99^th^ percentile E-field) were higher than the ROI electric field, this suggests that the tDCS montage delivers the maximal E-field outside of the cortical target. Alternatively, if the ROI electric field were higher than or equivalent to the 99^th^ percentile electric field, this suggests that the cortical target is located within the top 1% of activated voxels. For each participant, we plotted the motor ROI E-field (blue) and 99^th^ percentile E-field (red) and computed an intraclass correlation coefficient (ICC) as a statistical measure of similarity between the ROI and 99^th^ percentile E-fields in the five Round 1 models (bilateral M1, M1-SO, HD-tDCS, left right pad surround (LRPS)-tDCS, and anterior posterior pad surround (APPS)-tDCS)(**Supplementary Figure 2a-e**).

The ICC results consistently demonstrated that conventional electrode placements deliver maximal E-fields off-target whereas surrounding the intended target focuses the highest E-field within the motor cortex. For conventional bilateral M1 (**Supplementary Figure 2a**) and M1-SO tDCS (**Supplementary Figure 2b**), the 99^th^ percentile E-field was higher than the ROI E-field in 200 of 200 participants, suggesting that these conventional electrode placements consistently do not deliver the maximal E-field at the cortical motor target. While each comparison was statistically significant at the p < 0.001 significance level, the bilateral M1 ICC of 0.391 and M1-Supraorbital ICC of 0.241 fall within the ‘poor’ reliability range (considered to be an ICC < 0.5).

Regarding HD-tDCS, the 4 x 1 electrode strategy produced higher ROI than 99^th^ percentile E-field values in 200 of 200 participants due to the high focality of stimulation, with a ‘poor’ reliability ICC value of 0.430 (**Supplementary Figure 2c**). In contrast, for APPS-tDCS and LRPS-tDCS electrode montages, in which the electrodes surround the cortical target, there were statistically significant and ‘good’ similarity values between the 99^th^ percentile and ROI E-fields (APPS-tDCS ICC: 0.772; LRPS-tDCS ICC: 0.831), with the motor ROI values being higher than the whole brain 99^th^ percentile values in 200 of 200 participants for both electrode placements (**Supplementary Figures 2d-e**). Thus, the novel surround electrode montages of APPS-tDCS and LRPS-tDCS consistently deliver maximal E-fields on-target at the intended cortical target; the conventional placements of bilateral M1 and M1-SO focus the maximal amount of stimulation consistently off-target and therefore are inefficient methods of stimulating the intended cortical target region.

**Supplementary Figure 2:**

**
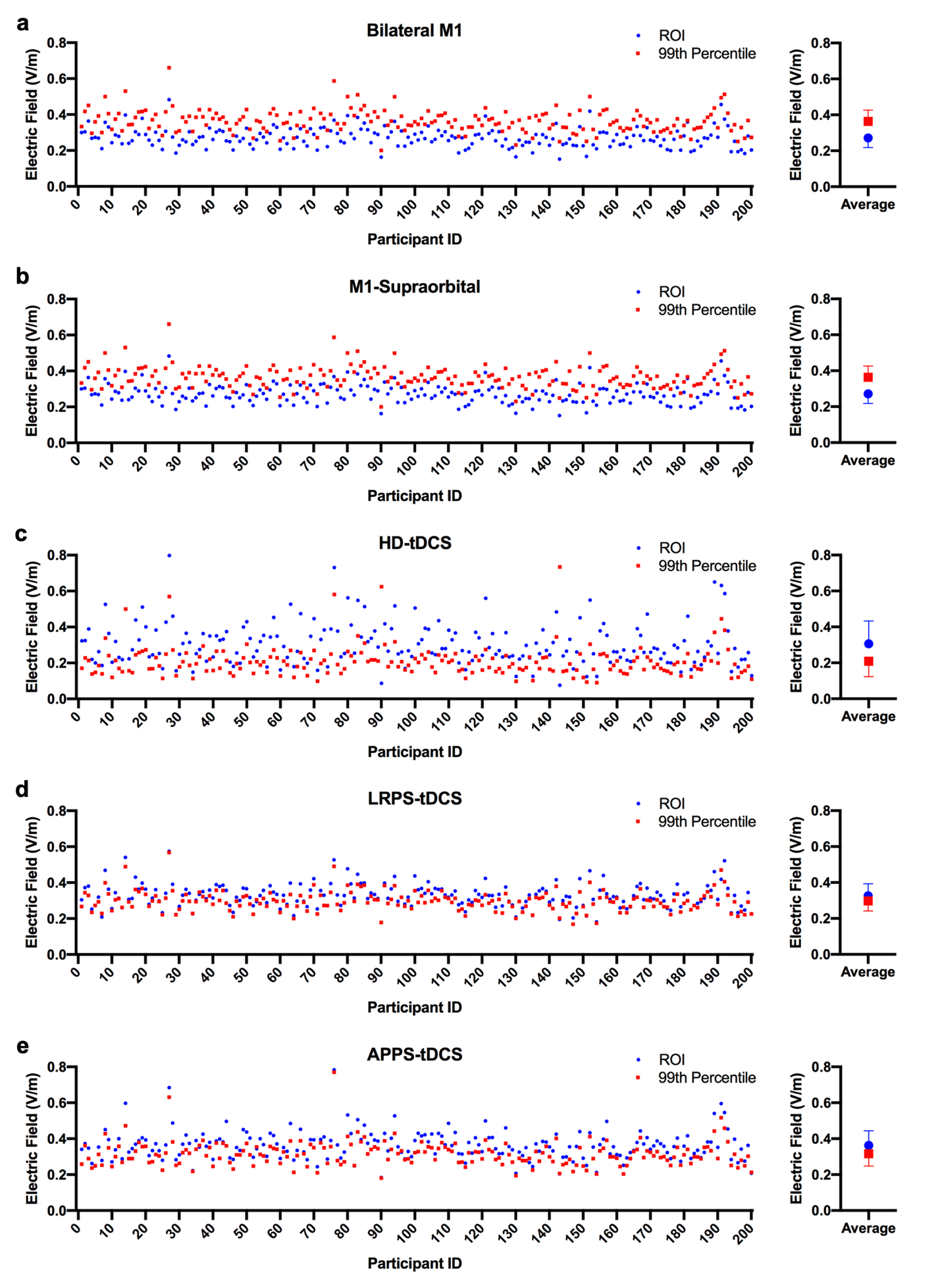
**

**Supplementary Figure 2: APPS-tDCS Induces the Maximal E-Field at the Intended Cortical Target While Conventional Electrode Placements Focus Stimulation Off-Target.** In this figure, we compared E-field magnitudes between the motor target (as assessed by the region of interest; ROI analysis), and the whole brain peak E-fields using the 99^th^ percentile threshold (top 1% of voxels included). If these measures are relatively similar, as assessed by an intraclass correlation coefficient (ICC), the whole brain peak E-field is situated at the cortical target ROI. If the 99^th^ percentile E-field exceeds that of the ROI, the maximal E-field is located outside of the cortical target. All ICCs were significant at the p < 0.0001 level, but with differing levels of consistency. For Bilateral M1 (**a**) and M1-SO (**b**), the 99^th^ percentile E-field consistently exceeded that of the ROI in each of the 200 participants, with ICCs of 0.391 and 0.241 respectively (‘poor’ reliability). For HD-tDCS (**c**) the ROI E-field was consistently higher than the 99^th^ percentile E-field, suggesting that HD-tDCS stimulated a cortical target with a very focal spread of stimulation, as the ROI E-field was in a more constrained space than that of the top 1% of voxels, but with a ‘poor’ reliability ICC of 0.430. For LRPS-tDCS (**d**) and APPS-tDCS (**e**), there was ‘good’ similarity between the ROI and 99^th^ percentile E-fields of 0.831 and 0.772 respectively, suggesting that the maximal E-field was located within the cortical target ROI in each of the 200 participants. These ICC analyses provide further evidence that placing electrode surrounding the cortical target, as we did in LRPS and APPS-tDCS, induces the maximal E-field at the target region. Graphs were generated in GraphPad PRISM 9.0.1 (<https://www.graphpad.com/scientific-software/prism/>).
